# Written Language Ability in Mandarin-Speaking Children with Cochlear Implants

**DOI:** 10.1155/2015/282164

**Published:** 2015-07-05

**Authors:** Che-Ming Wu, Hui-Chen Ko, Yen-An Chen, Yung-Ting Tsou, Wei-Chieh Chao

**Affiliations:** Department of Otolaryngology-Head and Neck Surgery, Chang-Gung Memorial Hospital, College of Medicine, Chang-Gung University, Linkou Branch, Taoyuan 333, Taiwan

## Abstract

*Objectives*. To examine narrative writing in cochlear implant (CI) children and understand the factors associated with unfavorable outcomes.* Materials and Methods*. Forty-five CI children in grades 2–6 participated in this study. They received CIs at 4.1 ± 2.1 years of age and had used them for 6.5 ± 2.7 years. A story-writing test was conducted and scored on 4 subscales: Total Number of Words, Words per Sentence, Morphosyntax, and Semantics. Scores more than 1.5 SD lower than the mean of the normal-hearing normative sample were considered problematic. Language and speech skills were examined.* Results*. Significantly more implanted students were problematic on “Total Number of Words” (*p* < 0.001), “Words per Sentence” (*p* = 0.049), and “Semantics” (*p* < 0.001). Poorer receptive language and auditory performance were independently associated with problematic “Total Number of Words” (*R* ^2^ = 0.489) and “Semantics” (*R* ^2^ = 0.213), respectively. “Semantics” problem was more common in lower graders (grades 2–4) than in higher graders (grades 5-6; *p* = 0.016).* Conclusion*. Implanted children tend to write stories that are shorter, worse-organized, and without a plot, while formulating morphosyntactically correct sentences. Special attention is required on their auditory and language performances, which could lead to written language problems.

## 1. Introduction

Writing could be one of the most complex tasks for all students, whether being normal-hearing or hearing-impaired students. For Mandarin Chinese students, who use a logographic orthography, they must know the correct spatial arrangements of strokes of a character, the conventions of punctuation, and the use of proper vocabulary and syntactical structures in order to write at a basic level. At a higher level, they have to choose topics and plan and organize their ideas [1].

It was indicated that early writing patterns during elementary school years are related to spoken language trends [2]. Deaf children were thus often found to use simple and short sentences with limited vocabulary and recurring phrases [3–7]. Their compositions were also reported to have fewer adjectives and adverbs [8] and more errors in the use of function words (e.g., omission or wrong usage of prepositions and pronouns) [9] due to the difficulty acquiring knowledge of syntactical and morphological structures [10, 11]. As a result, Antia et al. [12] reported that the mean written quotient of their deaf children was in the below-average range as compared to that of the hearing peers. Although the hearing-impaired students did improve their writing skills with age [1, 5], they made slower progress than the hearing children [5, 6, 13, 14], which could have an impact on their academic performance throughout the school years [15].

With the restoration of hearing via a cochlear implant (CI), it is expected that the improvements in spoken language may also lead to the improvements in other language skills such as writing. Spencer et al. [2] found that the children with CIs performed within 1 standard deviation (SD) of the normal-hearing age mates on the measure of writing accuracy. One study also indicated that the spelling skills of the implanted children aged between 6 to 12 years were comparable to those with normal-hearing who were matched for reading ability [16]. However, when compared to the age-matched children, a significant difference was found between the two groups of children. The CI children's performance on formulating sentences was found to fall behind the hearing peers as well, and they were reported to produce fewer words on the expository writing although no significant difference was noted in terms of total words per clause [2].

The problem with written language was not necessarily eased with age. It was found that high-school students with CIs also spelled significantly poorer than the hearing peers, and less than 50% of them scored within 1 SD of the hearing group on the expository writing task [17]. The age at implantation may also affect writing performance. Those who were implanted after the age of 4 years were reported to have difficulty with lower-level writing, that is, expression formation and productiveness, while those who received CIs before that age had problem with higher-level writing, that is, assisting key tone [18].

Nevertheless, to date, most of the studies on the written language skills in the implanted population use only subjects with an alphabetic language background, such as English. There is hardly any study investigating the patient group that uses Mandarin Chinese, a logographical language. While the basic unit for writing in alphabetic languages is segments [19, 20], it is the characters in Chinese (e.g., “馬”), each representing a syllable in sound (/ma3/) and a morpheme in meaning (“horse”). This means that the phoneme-grapheme correspondence in Chinese characters is not transparent. However, when children in Taiwan enter elementary school, they are compulsorily taught a system of alphabetic symbols called Zhu-Yin (including 37 symbols) to learn the pronunciation of Chinese characters [21]. These features of Chinese characters could result in different writing performance in Mandarin-speaking patient group from that of the alphabetic language users reported by previous studies.

Therefore, this study aimed (1) to examine the narrative writing performance in Mandarin-speaking elementary-school students with cochlear implants and (2) to understand the family/child-related, implant-related, and language-related factors associated with less favorable writing performance.

## 2. Patients and Methods

### 2.1. Participants

Forty-five prelingually deafened patients (20 boys, 25 girls) who received CIs in our center participated in this study. They all met the inclusion criteria: (1) the subjects were in grades 2–6 of elementary school; (2) the subjects did not have any developmental problems or additional handicaps (e.g., intellectual disability, attention deficit, language disorders, learning disability, and autism); (3) the subjects used oral communication and Mandarin Chinese as their major language; (4) the subjects went to mainstream schools and were not placed in the resource class; (5) the subjects' performance intelligence quotient [22, 23] was higher than 85. They were 8.0–13.3 years of age (mean = 10.6 ± 1.6) when taking the tests. They received the implantation at a mean age of 4.1 ± 2.1 years during years 2000–2010 and had used the implant for a mean duration of 6.5 ± 2.7 years.

The socioeconomic status (SES) of the family was determined based on the Hollingshead two-factor index of social status [24], which used a five-level item to rate the level of parents' educational background (1 = illiterate; 5 = with a graduate degree or above) and occupational status (1 = unskilled workers; 5 = higher executives/major professionals). The parents of the subjects were required to fill out a form to report the information.

All informed consents signed by participants and guardians were obtained before the test procedures. The study protocol was approved by the Institutional Review Board, Chang-Gung Memorial Hospital, Taoyuan, Taiwan.

### 2.2. Test Materials and Procedures

#### 2.2.1. Written Language Ability Measure

*(1) Written Language Ability Diagnostic Test for Children*. The test, designed based on the Myklebust Picture Story Language Test [25], is used to assess the written language skills of Mandarin-speaking students in elementary school [26]. The subjects were asked to write a story as long as possible by themselves about a given picture (see Figure 2). They were encouraged to write the entire article with Chinese characters, but Zhu-Yin symbols was also allowed in cases that they did not know how to write some of the characters. There was no time limit, and the picture was available throughout the test session. Their product was graded according to four subscales: (1) the subscale of Total Number of Words of the written story (“Total N Words”); (2) the subscale of number of words per sentence (“Words per Sentence”); (3) the “Morphosyntax” subscale that yielded a quotient computed from the number of incorrect language usages (including miswritten words, improper punctuations, and wrong dictions), which could be further classified as errors due to addition, omission, substitution, and transposition; (4) the “Semantics” subscale that rated the story by five levels according to its concreteness/abstractness (1 = nonsense; 2 = concrete description; 3 = concrete imagination; 4 = abstract description; 5 = abstract imagination). Higher scores indicated better skills. The raw scores were converted to standard* T* scores derived from a normative sample of 1800 normal-hearing students (mean = 50 ± 10) provided by the test developer [26]. The performance of our CI subjects was also compared to the normative sample and was classified as excellent (more than 2 SD higher than the normative mean), good (0.5 to 2 SD higher), normal (±0.5 SD of the normative mean), marginal (0.5 to 1.5 SD lower than the normative mean), or clinical (more than 1.5 SD lower). Marginal or clinical performances were considered unfavorable in this study, and thus those subjects that fell within these two ranges were defined as within the “problematic range” for further analysis. The validity and reliability of the test have been confirmed [26].

#### 2.2.2. Auditory Performance and Speech Intelligibility Measures

*(1) Categorical Auditory Perception (CAP) and Speech Intelligibility Rating (SIR) Scales*. The CAP and SIR scales are designed to assess deaf patients' auditory performance and speech production intelligibility, respectively (see Table 6). The CAP is a nonlinear hierarchical rating scale with 8 points (0 = unaware of environmental sounds; 7 = able to converse on the telephone with a familiar person). The SIR is a nonlinear scale that classifies children's speech production intelligibility into 5 levels (1 = unintelligible; 5 = easily understood by all listeners). The reliability of both scales has been confirmed [27–29].

#### 2.2.3. Language Skill and Speech Perception Measures

*(1) Test of Reading Comprehension*. This test evaluates the paragraph reading ability in elementary-school students [30] (Table 5). It includes 12 articles, each with 6–9 corresponding multiple-choice questions. The articles and the questions are shown only with Chinese characters, not accompanied by alphabetic Zhu-Yin symbols. The text remained available while answering, and there was no time limit. The number of correctly answered questions was turned into percentages to obtain overall scores. The reliability and validity of this test have been confirmed [30].

*(2) Graded Chinese Character Recognition Test*. This is a standardized test for assessing monosyllabic word identification in children in grades 1–9 [31]. The subjects need to identify 200 characters on the word list by writing down the Zhu-Yin symbols of each character. The raw scores were transformed to* T* scores according to the data of a grade-matched normative sample provided by the test developer [31].

*(3) Revised Primary School Language Assessment*. This test is designed to assess the language abilities in 6- to 12-year-old children from 4 aspects: receptive language, expressive language, voice and fluency, and articulation/tone error pattern (see the appendices of Wu et al. [32] for details about the test) [30]. Only receptive and expressive language subtests were included in this study, which were given orally to the subjects. Both tests deal with the semantic and pragmatic aspects of language use (i.e., understanding/expressing the meaning of a phrase or a sentence in a certain context). The raw scores were converted into* T* scores based on an age-matched normal-hearing normative sample (mean = 50 ± 10) [30].

*(4) Peabody Picture Vocabulary Test: Revised*. This test evaluates receptive vocabulary knowledge in children aged 3–12 years [33]. The students were presented with sets of four pictures and asked to point out one picture that best described the word spoken by the examiner, following basal and ceiling rules to administer the test.* T* scores (mean = 100 ± 15) were derived from the normal-hearing normative sample provided by the test developer [33].

*(5) Phonetically Balanced Word Perception Test*. This test uses 25 phonetically balanced monosyllabic words to test word perception ability [34]. The examiner spoke each word with mouth covered, and the subjects needed to verbally repeat the words they heard. They were scored based on the number of words they correctly repeated, which was converted into percentages for further analysis.

### 2.3. Statistical Analysis

The SPSS software (version 17.0; SPSS, Inc., Chicago, IL, USA) was employed to do the statistical analysis. The analysis of the written language ability was performed based on the ranges (normal range = 0; problematic range = 1) rather than on the* T* scores to make sure that we targeted on the patients who had problems with expressive writing. A chi-square goodness-of-fit test was used to compare the distribution of writing outcomes between our CI users and the normative sample. A Mann-Whitney* U* test was employed to make between-group comparison of test results. A binary logistic regression analysis was utilized to investigate the significance of child/family characteristics and language/speech skills in association with narrative writing problems. These variables of interest were split into two groups according to the medians for regression analysis. The chronological age and grade were not entered into the analysis because in this study* T* scores, which were derived from grade-matched normative sample, were used for evaluation. A *p* value of less than 0.05 was considered statistically significant.

## 3. Results

### 3.1. Narrative Writing Performance in Cochlear Implanted Children

The implanted subjects averagely produced 127.0 ± 69.3 words in their stories, each sentence with 9.5 ± 1.7 words (Table 1). The mean rate of making morphosyntactical mistakes in their production was 5.6% in total (Table 2), meaning that, in every 100 words, only 5.6 elements (characters or punctuations) were used incorrectly. Regarding “Semantics,” 20 subjects (44.4%) were at level 2 (concrete description), 14 (31.1%) at level 3 (concrete imagination), 5 at level 4 (abstract description), and 6 at level 5 (abstract imagination). None were regarded as producing nonsensical stories (i.e., at level 1; see Table 3).

**Figure 1:**
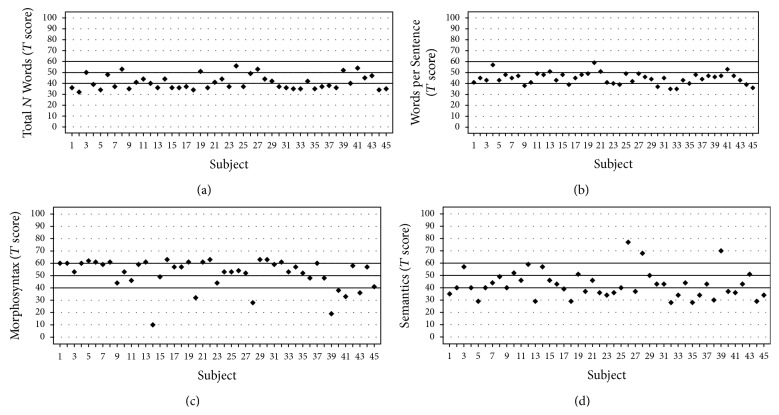
Individual standard scores for the four subscales of the Written Language Ability Diagnostic Test plotted for 45 children, with subscales including (a) “Total Number of Words,” (b) “Words per Sentence,” (c) “Morphosyntax,” and (d) Semantics. Horizontal lines indicate standard scores within 1 SD of the normal-hearing normative sample.

**Figure 2:**
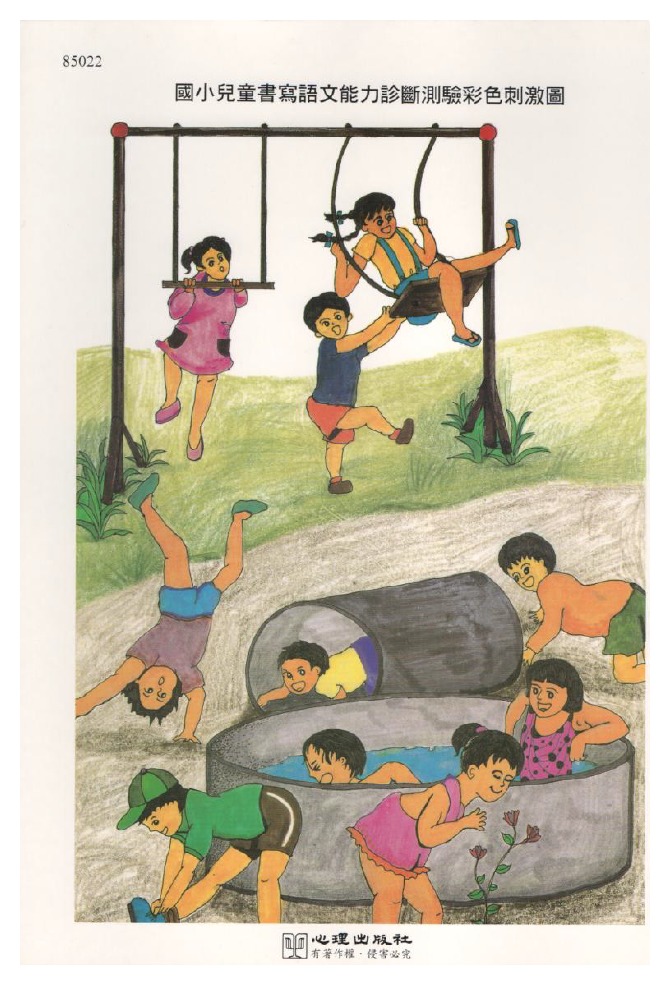
Stimulus picture for the Written Language Ability Diagnostic Test for children.

The CI subjects had* T* score of 40.9 ± 6.5, 44.7 ± 5.3, 51.6 ± 12.2, and 42.5 ± 11.3 on the subscales of “Total N Words,” “Words per Sentence,” “Morphosyntax,” and “Semantics,” respectively (see Table 1). The results suggest that their average* T* scores were within 1 SD of the mean of the normal-hearing normative sample (i.e., 50 ± 10, as provided by the test developer). 46.7%, 82.2%, 84.4%, and 57.8% of the subjects fell within the range of 1 SD from the normative mean of “Total N Words,” “Words per Sentence,” “Morphosyntax,” and “Semantics,” respectively (Figures 1(a)–1(d)).

However, there were significantly more CI students in the problematic range (i.e., more than 1.5 SD lower than the normative mean) on the subscales of “Total N Words” (*p* < 0.001), “Words per Sentence” (*p* = 0.049), and “Semantics” (*p* < 0.001; see Table 4). Actually, more than three-fourths of the CI subjects were in the problematic range on “Total N Words” subscale.

### 3.2. Clinical Factors Associated with Problematic Written Language

The factors related to child/family characteristics and language/speech skills were entered into a binary logistic regression analysis. The child/family characteristics included age at implantation, duration of implant use, SES, CAP, and SIR scores. The language-related parameters included paragraph reading, Chinese character recognition, receptive vocabulary, receptive language, expressive language, and word perception. Results showed that lower scores of receptive language (i.e., lower than the median score = 48.5) were independently associated with problematic performance on the “Total N Words” subscale (*p* = 0.026; odds ratio = 26.8; 95% confidence interval = 1.5–489.4;* R* ^2^ = 0.489). Lower scores of CAP (i.e., lower than the median score = 7) were independently associated with problematic “Semantics” (*p* = 0.035; odds ratio = 10.7; 95% confidence interval = 1.2–96.4;* R* ^2^ = 0.213).

### 3.3. Development of Written Language Skills during Elementary-School Years

Although chronological age and grade were not taken into concern in the regression analysis, we still would like to know how written language ability was developed during elementary-school years in the implanted children. Therefore, the subjects were split into two groups according to their grades (median = 4): the lower graders (those who are in grades 2–4; *n* = 24); and the higher graders (in grades 5-6; *n* = 21). The results showed that significantly more lower graders had problematic “Total N Words,” “Words per Sentence,” and “Semantics” compared to the grade-matched normative sample (see Table 4). Also, significantly more higher graders fell within the problematic range regarding their performances on “Total N Words” and “Semantics.” When compared the lower graders with those in the higher grades, significantly more lower graders were regarded as having problematic “Semantics” (*p* = 0.016), while no significant differences were noted between the two groups regarding the other three subscales (*p* > 0.05).

## 4. Discussion

Written language ability is essential for academic performance. Yet, it has never been carefully examined in prelingually deaf CI children with a Mandarin Chinese language background. Our preliminary results show that, compared to normal-hearing grade-matched children, significantly more implanted children have problematic performance on “Total N Words” and “Semantics” during narrative writing. Their receptive language skills and auditory performance have a close association with these problems, respectively.

CI children's narrative writing ability is examined from four perspectives, among which “Total N Words” seems to be the most problematic for the implanted students. Their production is significantly shorter than that of the normal-hearing children. This finding is in line with most of the previous studies on deaf and cochlear implanted children [2–6, 35]. It suggests that the productiveness is one of the most serious problems with deaf children's writing. This weakness, as our regression results show, is associated with the subjects' receptive language skills. That is, writing not only is about formulation of sentences but also is related to the ability to understand what is communicated orally. It is very likely because early writing patterns in deaf children follow spoken language trends during elementary-school years [2]. Spencer et al. [2] further indicate that the sentences composed by 9- to 15-year-old deaf children evolve in structure, from conjoined to embedded sentence structure. It shows that, with the development of spoken language, deaf children gradually learn to increase the complexity of form in writing. Therefore, specialists should not just focus on syntactical problems but also pay attention to the development of receptive language ability because it could be an underlying cause of their unproductiveness in writing.

However, although the implanted children are less productive, it seems that they have no difficulty forming morphosyntactically correct sentences. Actually, almost 80% of our subjects show normal or better-than-normal performance on the “Morphosyntax” subscale. Only a mean error rate of 5.6% was found in all subjects (Table 2), and their scores on this subscale are not significantly different from those of the normal-hearing. Unlike many past studies that indicate more errors with language use made by deaf or hard-of-hearing children than by normal-hearing ones [6, 9, 15], the present study shows that our Mandarin-speaking children with CIs have normal use of grammar at character and sentence levels.

This discrepancy occurs not only because our subjects have used the CIs for a long duration of 6.5 years averagely, but also very likely as a result of the logographic orthography used in Mandarin Chinese and the type of test applied in the current study. The written language test administered in this study focuses more on the morphology than the syntax on the “Morphosyntax” subscale. That is, it examines, firstly, whether the subjects make mistakes in words and punctuations and secondly, whether the error with words/punctuations is made because of addition, omission, substitution, or transposition. It remains unknown how well or erroneously these CI children manipulate different lexical categories, such as nouns, verbs, and adverbs, and different structures, such as interrogative and coordinate sentences.

Moreover, when these Mandarin-speaking students write in Chinese, they engage with logographemes rather than phonemes, so their auditory performance may not impede “spelling,” resulting in a mean miswritten word rate of only 0.8% (see Table 2). Although CI students are not necessarily bad “spellers” as Geers and Hayes [17] claim them to be (the study used English-speaking high-school students with CIs), it is indeed a limitation that we did not include a Zhu-Yin spelling test to analyze their phonological ability, which was due to lack of a standardized test for this purpose. Therefore, a test that more comprehensively examines different aspects of written language is certainly needed for Mandarin-speaking patients. It requires further investigations to develop such a test in the future.

Another problem with the stories written by our CI students is that they tend to engage only with concrete description. That is, more than two-fifths (44%) of the subjects write a story that does not have a main point and is not well organized or consistent (Table 3). Also, these children merely describe the objects they see in the given picture without mentioning the actions or feelings of the people in the picture. Only 13% of the children write a story that has a plot (Level 5). This weakness in writing is also found in English-speaking students with deafness who are indicated to have lower ratings of various aspects of semantics [13] and elaborate their ideas less fully [6] than the normal-hearing students. Geers and Hayes [17] also found that their CI students in high school obtained the lowest score on “organization” in expository writing. Fortunately, it is possible that the implanted children improve their semantic performance with age as our result shows that less higher graders (in grades 5-6) are deemed as having problematic “Semantics” compared to the lower graders (in grades 2–4) in spite of the fact that the performance of the higher graders is still worse than their normal-hearing grade mates.

The lack of capability of developing a story and expressing themselves fully may be associated with their less satisfactory auditory performance, considering that the CAP score is an independently associated factor of “Semantics” subscale in this study. It suggests that the ability to express oneself and organize a story in writing may develop with the increase in auditory experiences. Special trainings may be needed to improve their narrative writing ability because children with CIs seem to develop such ability at a slower rate than the normal-hearing ones, resulting in their lower scores on this subscale throughout the elementary-school years.

It has to be noted, however, that the structure organization of the story was not taken into concern in the current study. We focus on the semantic and morphosyntactical levels to understand the basic writing ability in students with CIs. Yet, narrative production does have a close association with the ability of linguistic structure organization, that is, the ability to concatenate different parts into a story (e.g., a beginning, a conflict and a corresponding resolution, and an ending), and the productiveness of written narratives is related to the production of oral narratives, as our result shows. It is therefore of particular interest to learn the correlation between oral and written narratives on a discursive level of language in future studies.

## 5. Conclusions

Our preliminary results show that children with CIs tend to write shorter stories which are not well organized and without a plot. These weaknesses in narrative writing are associated with their poorer receptive language skills and auditory performance. However, their ability to formulate morphosyntactically correct sentences is as good as the normal-hearing grade mates. Specialists thus are suggested not to focus only on syntactical problems but also on the development of auditory perception and receptive language for they could be the underlying causes of the writing problems. Also, a test that more thoroughly examines different aspects of written language needs to be developed in the future in order to better evaluate the written language problems in Mandarin-speaking patients with CIs.

## Figures and Tables

**Table 1:** Results of Written Language Ability Diagnostic Test in the children with cochlear implants.

Test results	Range	Mean ± SD
Total Number of Words	22.0–314.0	127.0 ± 69.3
Total Number of Words (*T* score)	32.0–56.0	40.9 ± 6.5
Total number of sentences	3.0–32.0	13.2 ± 6.8
Words per Sentence	6.6–13.0	9.5 ± 1.7
Words per Sentence (*T* score)	35.0–59.0	44.7 ± 5.3
Morphosyntax (*T* score)	10.0–63.0	51.6 ± 12.2
Semantics (*T* score)	28.0–77.0	42.5 ± 11.3

**Table 2:** Error rates of each of assessment items on the “Morphosyntax” subscale.

	Addition	Omission	Substitution	Transposition	Total
Diction (%)	1.06	0.97	0.55	0.09	2.68
Miswritten words (%)	0.06	0.07	0.63	0.00	0.77
Punctuation (%)	0.09	1.35	0.74	n/a	2.18

Total (%)	1.21	2.40	1.92	0.09	5.62

**Table 3:** Description of each level on the “Semantics” subscale and number (%) of cochlear implanted patients at each level.

Semantic level	Raw score		Number of children (%)
(1) Nonsense	0	Daubing; nonsensical phrases; unrelated subject to the given picture.	0 (0)

(2) Concrete description	1	Using a series of nouns.	3 (6.7)
	2	Using verb-noun structure to signal actions; using only one verb.	6 (13.3)
	3	Using nouns, verbs (more than one), and adjectives; able to make categorization.	11 (24.4)

(3) Concrete imagination	4	With a main point and a structure; description of actions and feelings of people in the picture.	7 (15.6)
	5	With a consistent main point throughout the story and a better organized structure; description of actions, feelings, and relations of people in the picture.	7 (15.6)

(4) Abstract description	6	With some plot; describing people in the picture as a group.	4 (8.9)
	7	With a setting; the entire story being set in one single context (e.g., family, school, playground, and park); structuring the story based on what the storyteller feels and perceives.	1 (2.2)

(5) Abstract imagination	8	With a plot, which is developed based on the picture; description of how people in the story feel and their motivations of taking certain actions.	3 (6.7)
	9	Longer story with more details and a more complicated plot; able to show causal relationship; description of events that are imaginary or may happen in the future.	1 (2.2)
	10	Description of abstract concepts that are beyond the picture contents; writing a prose/essay, exposition or fable rather than a story; expressing concerns about moral issues or welfare of human beings.	2 (4.4)

**Table 4:** Percentage of patients in the normal range and the problematic range on the four subscales of the Written Language Ability Diagnostic Test and a comparison between the distribution of cochlear implanted subjects and that of the normal-hearing grade-matched normative sample using chi-square goodness-of-fit test.

Subjects for comparison	Range	Total *N* Words (%)	Words per Sentence (%)	Morphosyntax (%)	Semantics (%)
All subjects	Normal	24.4	55.6	77.8	62.2
	Problematic	75.6	44.4	22.2	37.8
	*p* value^a^	<0.001	0.049	0.208	<0.001

Lower graders	Normal	33.3	45.8	70.8	50.0
	Problematic	66.7	54.2	29.2	50.0
	*p* value^a^	<0.001	0.01	0.85	<0.001

Higher graders	Normal	14.3	66.7	85.7	76.2
	Problematic	85.7	33.3	14.3	23.8
	*p* value^a^	<0.001	0.809	0.099	0.002

^a^The performances of the CI subjects (all, lower graders, and higher graders) on “Total N Words,” “Words per Sentence,” “Morphosyntax,” and “Semantics” were compared to a normal-hearing grade-matched normative sample, where 30.9%, 30.9%, 30.9%, and 6.7% of the students were in the problematic range on each subscale, respectively.

**Table 5:** Demographical data of the cochlear implanted subjects and the outcomes of language skill and speech perception measures.

Parameters	Mean ± SD	Median
Child/family-related		
Age at implantation (years)	4.1 ± 2.1	3.3
Duration of CI use (years)	6.5 ± 2.7	6.9
Grade	4.2 ± 1.6	4.0
SES^a^	2.3 ± 0.6	2.0
CAP	6.2 ± 0.5	6.0
SIR	4.7 ± 0.6	5.0
Language/speech-related		
Paragraph reading (%)	59.9 ± 19.6	60.0
Word recognition (*T* score)	51.4 ± 12.7	52.0
Receptive language (*T* score)	49.0 ± 12.6	48.5
Expressive language (*T* score)	52.4 ± 11.8	52.0
Receptive vocabulary	90.0 ± 13.5	90.0
Word perception (%)	85.7 ± 13.0	92.0

CI: cochlear implant; SES: socioeconomic status; CAP: Categorical Auditory Performance; SIR: Speech Intelligibility Rating.

^a^SES of the family (1 = low SES; 5 = high SES) was determined based on the Hollingshead two-factor index of social status that referenced to the parents' occupational status (1 = unskilled workers; 5 = higher executives/major professionals) and educational level (1 = illiterate; 5 = with a graduate degree or above).

**Table 6:** Criteria of Categorical Auditory Performance and Speech Intelligibility Rating scales.

Rating	Criteria of Categorical Auditory Performance	Criteria of Speech Intelligibility Rating
7	Use of telephone with known listener	n/a
6	Understanding of conversation without lip-reading	n/a
5	Understanding of common phrases without lip-reading	Connected speech is intelligible to all listeners. Child is understood easily in everyday contexts
4	Discrimination of some speech sounds without lip-reading	Connected speech is intelligible to a listener who has a little experience of a deaf person's speech
3	Identification of environmental sounds	Connected speech is intelligible to a listener who concentrates and lip-reads
2	Response to speech sounds	Connected speech is unintelligible. Intelligible speech is developing in single words when context and lip-reading cues are available
1	Awareness of environmental sounds	Connected speech is unintelligible. Prerecognizable words in spoken language, primary mode of communication may be manual
0	No awareness of environmental sounds	n/a

n/a = not applicable.
